# Efficacy and safety of remifentanil for endoscopic ultrasound-guided tissue acquisition: a single center retrospective study

**DOI:** 10.1007/s00464-021-09006-8

**Published:** 2022-01-18

**Authors:** Yueh-Juh Lin, Yi-Chia Wang, Hui-Hsun Huang, Chi-Hsiang Huang, Pei-Lin Lin

**Affiliations:** 1 Department of Cardiology, En Chu Kong Hospital, New Taipei City, Taiwan; 2 Department of Anesthesiology, National Taiwan University Hospital, No. 7, Chung-Shan South Road, Taipei, 100225 Taiwan

**Keywords:** Remifentanil, Endoscopic ultrasound, Target-controlled infusion, Bispectral Index monitoring

## Abstract

**Background:**

Remifentanil is a rapid onset and rapid recovery opioid. The combination of remifentanil and propofol for deep sedation decreases the incidents of movement, cough, and hiccup. We evaluated the efficacy and safety of remifentanil during endoscopic ultrasound-guided tissue acquisition.

**Methods:**

We retrospectively reviewed patients in whom endoscopic ultrasound-guided tissue acquisition was performed for solid mass lesions of the upper gastrointestinal tract and adjacent organs. All patients were premedicated with midazolam (2 mg), and target-controlled infusion of propofol, opioid, and Bispectral Index (BIS) monitoring were administered as necessary to maintain moderate-to-deep sedation. The opioids used were a bolus of alfentanil or remifentanil infusion. The discharge time, consumption of propofol and opioid, adverse events, diagnostic accuracy, and sensitivity and specificity for malignancy, were compared.

**Results:**

Tissue acquisition was achieved in 123 patients (alfentanil group, *n* = 64; remifentanil group, *n* = 59). The discharge time of the remifentanil group (16.5 ± 3.2 min) was significantly shorter than that of the alfentanil group (19.0 ± 4.9 min, *P* = 0.001). The consumption of propofol, adverse events, diagnostic accuracy, sensitivity, and specificity for malignancy in the alfentanil group were not significantly different from those in the remifentanil group.

**Conclusions:**

Use of alfentanil or remifentanil for target-controlled infusion of propofol–BIS monitoring can provide good sedative and diagnostic quality for endoscopic ultrasound-guided tissue acquisition. However, remifentanil resulted in faster recovery than alfentanil.

Endoscopic ultrasound-guided tissue acquisition is the preferred modality for diagnosing and staging neoplasm in the gastrointestinal tract and adjacent organs, such as the pancreas, bile duct, submucosal lesions, adrenal glands, liver, retroperitoneal masses, lymph nodes, and posterior mediastinum [1]. Anesthesiologist-directed anesthesia improves the success rate of the endoscopic ultrasound procedure [2]. Compared with standard endoscopic ultrasound, endoscopic ultrasound-guided tissue acquisition is more invasive, unpredictable, and time consuming. Therefore, it is essential to select sedatives with a rapid onset, short half-life, and few adverse events.

Meta-analyses have demonstrated that propofol for advanced endoscopic procedures is associated with shorter recovery time and better sedation quality and amnesia level without an increased risk of cardiopulmonary complications [3]. However, propofol–opioid dosing regimens lead to better sedative conditions for esophageal instrumentation than propofol alone [4]. The rapid onset and short half-life of remifentanil, a potent opioid, facilitate the titration of drug dose according to each patient’s needs [5]. The combination of remifentanil and propofol for deep sedation decreases the incidents of movement, cough, and hiccup during colonoscopy [6]. A few studies have mentioned the impact of remifentanil during advanced gastrointestinal endoscopy [7–13].

Target-controlled infusion of propofol with Bispectral Index (BIS) monitoring and bolus of alfentanil allows a lower propofol infusion rate and a higher satisfaction for endoscopists during advanced gastrointestinal endoscopy [14]. Nieuwenhuijs et al. reported that when infusions of remifentanil and propofol were used together, the depressant effects on blood pressure and heart rate were additive, while the depressant effects on respiration was strikingly synergistic [15]. The modeled context-sensitive half-time for a 3-h infusion of alfentanil is 50–55 min and is 3 min for remifentanil [16]. Bolus of alfentanil and infusion of remifentanil are commonly used method of administration. However, the differences in outcome with the use of different opioids for endoscopic ultrasound-guided tissue acquisition remain unclear. This study aimed to evaluate the efficacy and safety of remifentanil under target-controlled infusion of propofol and BIS monitoring during endoscopic ultrasound-guided tissue acquisition. The primary outcome was discharge time. The secondary outcomes were consumption of propofol and opioid, adverse events, diagnostic accuracy, and sensitivity and specificity for malignancy.

## Methods

This retrospective study was approved by our Institutional Review Board (No: 201911110RINB). All endoscopic ultrasound-guided tissue acquisition examinations were performed by experienced endoscopists and cytopathologists at a university-affiliated tertiary care teaching hospital. We included patients who underwent endoscopic ultrasound-guided fine-needle aspiration and/or biopsy of solid mass lesions of the upper gastrointestinal tract and adjacent organs under midazolam/target-controlled infusion propofol/BIS monitoring with alfentanil or remifentanil from July 2017 to September 2019; these solid mass lesions were detected through imaging modalities, including ultrasound, computed tomography, and magnetic resonance imaging.

Exclusion criteria were age < 20 years, pure cystic lesion without solid component as target lesion for sampling, and lack of adequate follow-up data. Data collection was performed using the hospital electronic medical record system (National Taiwan University Hospital). The anesthetic records included the American Society of Anesthesiologists (ASA) physical status, medication, adverse events, anesthetic time, and discharge time. The electronic endoscopy database included procedure time, largest dimension of the lesion, and lesion location; moreover, the number of passes, final pathology, procedural variables, complications, and follow-up pathology were obtained. The sensitivity and specificity for malignancy, and diagnostic quality for endoscopic ultrasound-guided tissue acquisition examinations are compared. The pathological diagnosis with endoscopic ultrasound was categorized as inadequate, benign, atypical, suspicious, or malignant. Specimens categorized as suspicious for malignancy were considered diagnostic in patients with a high clinical suspicion of malignancy. Atypia was considered non-diagnostic. Diagnostic accuracy was calculated and compared with the standard diagnosis in (1) operated patients, based on the diagnosis of the surgically resected specimen, and (2) non-operated patients, based on the conclusions of the diagnostic work-up (combined outcomes of additional tissue sampling and imaging studies) and confirmed with a compatible clinical disease course of ≥ 6 months.

### Monitoring and medication

All patients were continuously monitored for heart rate, peripheral oxygen saturation, and electrocardiographic changes. Blood pressure was assessed automatically at 5-min intervals, and all vital signs were recorded at 5-min intervals. All patients were monitored using a BIS™ Quatro 4-electrode Sensor connected to the BIS™ VISTA monitoring system. In the alfentanil group, patients were premedicated with midazolam (2 mg) and alfentanil (0.4 mg) before endoscope insertion. The study protocol specified that patients who achieved the targeted depth of sedation but exhibited evidence of hypertension were given an additional 0.25 mg of alfentanil. In the remifentanil group, patients were premedicated with midazolam (2 mg) and a remifentanil infusion at 0.125 μg kg^−1^ min^−1^ for 2 min (remifentanil concentration: 12.5 μg ml^−1^), followed by a continuous infusion of 0.025–0.1 μg kg^−1^ min^−1^, before advanced gastrointestinal endoscopy. In both groups, the initial target blood concentration of target-controlled infusion propofol was set at 1.0 μg ml^−1^ with adjustments of 0.2 μg ml^−1^. The levels of sedatives were adjusted as necessary to maintain moderate-to-deep sedation and were monitored according to BIS scores of 60–80 [14, 17]. Anesthesia included midazolam (5 mg; F. Hoffmann-La Roche, Cenexi SAS, Fontenay-sous-Bois, France), alfentanil (1 mg; Hameln pharmaceuticals Gmbh, Hameln, Germany), propofol (200 mg; Fresenius Kabi Austria GmbH, Graz, Austria), and remifentanil (2 mg; Laboratorio Reig Jofre SA, Barcelona, Spain). Propofol was infused through an Injectomat® TIVA Agilia syringe pump (Fresenius vial, Brezins, France) through a target-controlled infusion system using the Schnider model.

### Statistical analysis

All statistical analyses were conducted using SPSS for Windows v22 (IBM Corp., Armonk, NY, USA). Data are represented as mean ± standard deviation. The *χ*^2^ test or Fisher’s exact test were used to compare categorical variables and the Student’s *t* test and Wilcoxon rank-sum test for continuous variables. Results with *P* < 0.05 were accepted as statistically significant. Diagnostic performance characteristics, including sensitivity, specificity, positive predictive value, negative predictive value, and accuracy, were calculated. Sensitivity for diagnosing malignancy, specificity for diagnosing benign disease, and diagnostic accuracy were defined as the sum of true positive and true negative values divided by the number of patients.

## Results

**Fig. 1:**
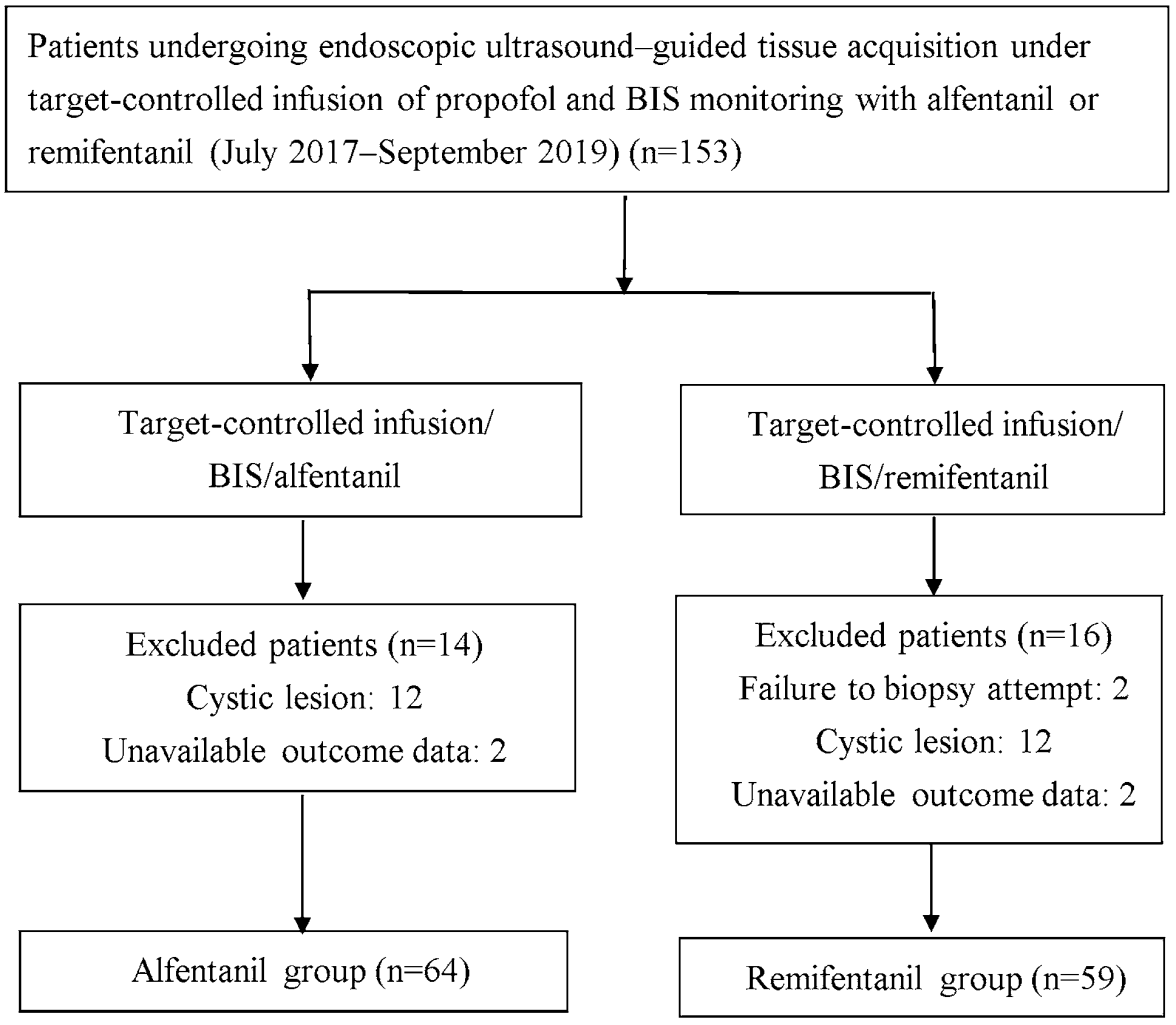
Flowchart of study selection. BIS, Bispectral index

Initially, 153 patients were enrolled (Fig. 1). Thirty patients were excluded for the following reasons: (i) failure to reach the target lesion due to surgical anatomical alterations (one patient with total gastrectomy with Roux-en-Y reconstruction and another patient with subtotal gastrectomy with Billroth II reconstruction, *n* = 2), (ii) pure cystic lesion (*n* = 24), (iii) lack of outcome data (*n* = 4). The data of the remaining 123 patients with solid mass were analyzed. Under midazolam/target-controlled infusion propofol/BIS monitoring, 64 patients received alfentanil and 59 received remifentanil. Their demographic characteristics are presented in Table 1. No significant between-group differences were noted in sex, age, body weight, body height, ASA physical status, tumor location, tumor size, or number of passes.

**Table 1:** Patient demographics

	Alfentanil group	Remifentanil group	*P* value
Sex (M:F), *n*	40:24	32:27	0.353
Age, mean (SD), years	65.1 (11.5)	61.3 (13.2)	0.90
Height, mean (SD), cm	162.8 (9.0)	162.7 (10.0)	0.943
Body weight, mean (SD), kg	61.9 (14.0)	61.6 (12.0)	0.889
ASA class, *n*			0.400
1	1	1	
2	43	44	
3	20	13	
4	0	1	
Lesion location, *n*			0.326
Head	23	18	
Neck	2	7	
Uncinate	1	3	
Body	23	10	
Tail	11	7	
Other organ	4	14	
Tumor size (largest dimension, cm), *n*			0.930
< 2 cm	13	12	
2–4 cm	30	27	
> 4 cm	21	20	
Number of passes, mean (SD)	3.8 (1.3)	4.0 (1.8)	0.510

*M:F* male:female, *n* numbers of patients

Anesthetic data are presented in Table 2. Total propofol dosage, duration of anesthesia, propofol infusion rate, and total procedure time were similar between the two groups. Four patients received an additional bolus of alfentanil. The remifentanil group were discharged significantly earlier than the alfentanil group (*P* = 0.001). The endoscopic records indicated one case of mild hemorrhage in the alfentanil group, but this did not interfere with the tissue sampling. No patient required a bolus of norepinephrine or an Ambu bag during the procedure.

**Table 2:** Anesthetic data for patients undergoing endoscopic ultrasound-guided tissue acquisition under target-controlled infusions of propofol and Bispectral Index (BIS) monitoring using bolus of alfentanil or remifentanil infusion

	Alfentanil group	Remifentanil group	*P* value
Total anesthetic time (min)	51.0 (18.5)	56.8 (19.6)	0.10
Total procedure time (min)	43.5 (17.6)	47.7(17.8)	0.19
Total propofol dosage (mg)	264.5 (161.4)	257.2(110.9)	0.77
Propofol infusion rate (mg kg^−1^ h^−1^)	4.92 (1.80)	4.57(1.52)	0.25
Remifentanil infusion rate (μg kg^−1^ min^−1^)		0.0465(0.0125)	
Discharge time (min)	19.0 (4.9)	16.5 (3.2)	0.001*
Adverse events, *n*			
Immediate bleeding	1	0	1
Norepinephrine	0	0	
Ambu bag	0	0	

All data are accompanied by their means (SD) and number of patient. *P < 0.05

*min* minutes, *h* hours, *n* number of patient

Diagnostic performance of endoscopic ultrasound-guided tissue acquisition is presented in Table 3. One unsatisfactory specimen was found in the remifentanil group due to duodenal scar and stricture. The pathological diagnostic accuracy regarding tumor mass was 93.8% (60/64) and 93.2% (55/59) in the alfentanil and remifentanil groups, respectively. No between-group differences were noted in sensitivity, specificity, positive predictive value, negative predictive value, or accuracy.

**Table 3:** Diagnostic performance for patients undergoing endoscopic ultrasound-guided tissue acquisition under target-controlled infusions of propofol and Bispectral Index (BIS) monitoring using alfentanil or remifentanil

	Alfentanil group	Remifentanil group	*P* value
Sensitivity (%)	92.2 (81.1, 97.8)	93.8 (82.8, 98.7)	1.00
Specificity (%)	100.0 (75.3, 100.0)	90.9 (58.7, 99.8)	0.46
Positive predictive value (%)	100.0	97.8 (87.4, 99.7)	0.50
Negative predictive value (%)	76.5 (55.9, 89.3)	76.9 (52.3, 91.0)	1.00
Accuracy	93.8 (84.8, 98.3)	93.2 (83.5, 98.1)	1.00

Values in brackets are 95% confidence interval

*P* value is based on Fisher’s exact test

## Discussion

In this study, the anesthetic safety of remifentanil was comparable to those of alfentanil during endoscopic ultrasound-guided tissue acquisition of solid mass. The discharge time was significantly shorter in the remifentanil group than in the alfentanil group. Although remifentanil has a faster onset and shorter duration of action than alfentanil and facilitated dose titration to the desired degree of effect, these characteristics failed to increase its diagnostic accuracy compared with alfentanil during endoscopic ultrasound-guided tissue acquisition.

As the complexity of endoscopy and proportion of aging with comorbidity increase, endoscopists may fail to divide their attention between performing the procedure and maintaining the sedation, particularly during endoscopic ultrasound-guided tissue acquisition. Remifentanil and alfentanil are two opioids with relatively short duration of action. Remifentanil has a very short terminal half-time and is independent of the duration of infusion, whereas prolonged administration of alfentanil results in a longer elimination half-life [18, 19]. Although remifentanil administered through target-controlled infusion resulted in a lower incidence in apnea compared with manually controlled infusion in patients undergoing colonoscopy, no differences were noted in the incidence of cough, excitatory movement, and hiccups between the two infusion protocols [6]. The target-controlled infusion mode lacks the bolus mode of administration, making purposeful and non-purposeful movements more obvious than in the manually controlled infusion mode. Therefore, we chose low-dose remifentanil infusion and alfentanil intermittent bolus to compare the impact during endoscopic ultrasound-guided tissue acquisition. Our data indicated rapid recovery in the remifentanil group. However, continuous remifentanil infusion did not decrease total propofol consumption, which is consistent with the findings of Wang et al. [20].

Glass et al. demonstrated that when ventilatory depression is used as a measure of opioid effect, remifentanil is approximately 40 times more potent than alfentanil [21]. The incidence of hypoxemia was more than three times higher in patients sedated with propofol and remifentanil than in those sedated with propofol and alfentanil. Therefore, remifentanil is not recommended for patient-controlled sedation during ERCP [10]. However, no differences were observed in adverse events between the two groups in our study. Inappropriate mode of administration, drug concentration, airway care, and differences in procedures may affect the outcomes. The remifentanil administered was diluted to a concentration of 12.5 μg ml^−1^, which is lower than the commonly recommended final concentration of 25 μg ml^−1^ for monitored analgesia care [22]. Target-controlled infusion of propofol and remifentanil infusion were administered proximally through an intravenous line to decrease the incidence of remifentanil- or propofol-induced cardiovascular instability and respiratory depression when other medications were administered (e.g., hyoscine injection and contrast agent). A study suggested the combination of low remifentanil and high propofol concentration to avoid intolerable ventilatory depression in patients undergoing moderate-to-deep sedation for esophageal instrumentation [23]. In addition, premedication with a low dose of midazolam reduces the initial propofol dose needed, thus potentially decreasing the incidence of propofol-induced cardiovascular instability and respiratory depression. Therefore, remifentanil is safe for endoscopic ultrasound-guided tissue acquisition if a low concentration, low dose, and manually controlled infusion mode are used.

A retrospective study indicated that the use of general anesthesia was associated with increased diagnostic yield (83% vs. 73% without general anesthesia) when performing endoscopic ultrasound fine-needle aspiration of pancreatic masses [24]. In the present study, we presented the diagnostic performance when using different opioids: the choice of alfentanil or remifentanil had no impact on diagnostic accuracy (93.8% vs. 93.2%) under target-controlled infusion of propofol with BIS monitoring. Both drugs provided good diagnostic and sedative quality. Remifentanil can be titrated as necessary to meet the patients’ hemodynamic and respiratory needs. Its use may allow the adjustment of patients’ respiratory rate and help endoscopists manage difficult cases of endoscopic ultrasound-guided tissue acquisition due to critical interventional structures (e.g., blood vessels and ducts) near the needle path. However, in cases of severely altered anatomy, such as total gastrectomy with Roux-en-Y reconstruction and subtotal gastrectomy with Billroth II reconstruction, the use of remifentanil did not help.

Our study has some limitations. First, the retrospective design meant that some confounding factors may have remained. Second, inability to blind the endoscopist to the type and size of the needle used, rapid on-site evaluation, sampling technique, and specimen handling and processing may have influenced the diagnostic outcome when using remifentanil. Third, our study sample size was not determined a priori; therefore, differences in outcomes might be underestimated due to insufficient sample size. Future large-scale prospective randomized controlled studies are warranted.

The cost for remifentanil is NT$513 (approximately US$18.32) for a 2-mg vial, and alfentanil costs NT$188 (approximately US$6.71) per 2-ml ampoule (containing 1 mg of the drug). Often, the entire ampoule of alfentanil is enough for one patient. Thus, remifentanil costs NT$325 (approximately US$11.61) more than alfentanil, but the difference in cost may potentially be offset by the extra time needed to monitor and care for the patients receiving alfentanil. In general, the content of one remifentanil vial is for single use only. However, remifentanil is stable for 24 h at room temperature after reconstitution and further dilution [22]. In practice, clinicians can use it for multiple patients in a given treatment day by drawing it using different sterile syringes from the same vial, thereby reducing waste. Because of these reasons, the drug cost in the remifentanil group may not exceed that in the alfentanil group.

In conclusion, we demonstrated that, when combined with low-dose midazolam, target-controlled infusion of propofol, and BIS monitoring, remifentanil and alfentanil were equally effective in terms of anesthetic performance, safety, and diagnostic accuracy during endoscopic ultrasound-guided tissue acquisition; the only difference was an earlier discharge in the remifentanil group. Thus, remifentanil administration resulted in faster recovery than did alfentanil.
